# Assessment of Surgical Outcome in Three- and Four-Part Proximal Humerus Fracture Treated With Proximal Humerus Internal Locking System (PHILOS) Plate Versus Neer’s Prosthesis in Elderly Patients

**DOI:** 10.7759/cureus.21857

**Published:** 2022-02-03

**Authors:** Sagar Gurnani, Tushar Pisal, Mukesh O Phalak, Tushar Chaudhari, Shivam Patel, Parth Yadav, SK Mizanur

**Affiliations:** 1 Orthopaedics, Dr. D. Y. Patil Medical College, Hospital & Research Centre, Pune, IND; 2 Orthopedics and Traumatology, Spine Surgeon, Dr. D. Y. Patil Medical College, Hospital & Research Centre, Pune, IND; 3 Orthopaedic Surgery, Dr. D. Y. Patil Medical College, Hospital & Research Centre, Pune, IND

**Keywords:** radiological outcome, philos plate, constant-murley score, neer’s prosthesis, proximal humerus fracture

## Abstract

Background: With an incidence of 50% of humerus fractures, proximal humerus fractures (PHFs) can significantly impact one’s quality of life. Moreover, management of highly comminuted or displaced PHFs poses a significant challenge amongst elderly population due to poor bone quality. Prosthetic replacement of humeral head or its stabilization using external plates is a commonly employed intervention for treating three- and four-part PHFs. Thus, these two methods were compared in this study to identify a preferable intervention.

Methods: Patients were randomly divided into two groups to receive proximal humerus internal locking system (PHILOS®, Synthes, Switzerland) plating and Neer’s hemiarthroplasty. The deltopectoral approach was deployed as the surgical method. Their surgical outcome was assessed from functional range of motion (ROM) and Constant-Murley scores at regular intervals of three, six, twelve, and twenty-four weeks.

Results: Twenty patients were divided into two groups who received PHILOS^®^ plating and Neer’s hemiarthroplasty, averaged 67.2 years and 72.8 years. The ROM pertaining to flexion, extension, abduction, internal rotation, and external rotation for individuals with PHILOS^®^ plating was 20%, 12.5%, 14.7%, 11.5%, and 18.5% higher than those who received Neer’s hemiarthroplasty. Moreover, the Murley score was also 8.7 units higher for individuals with PHILOS^®^ plating.

Conclusions: Prognosis following PHILOS® plate osteosynthesis had a better overall outcome than hemiarthroplasty with Neer’s prosthesis. Although hemiarthroplasty yielded a consistent functional outcome, PHILOS® plate osteosynthesis tends to restore a greater ROM. Thus, PHILOS® plating is recommended as the suitable method to manage three- and four-part PHF for people above fifty-five years of age.

## Introduction

Proximal humerus fracture (PHF) is the third most common fracture amongst older age (>65 years) and accounts for 4%-5% of all fracture types [1-3]. Even low-velocity injuries like a simple fall in the elderly population can cause PHF; thus, its incidence increases to 80% with age [3]. Half of the humerus fractures are PHF, and most PHFs are due to indirect injury, i.e., fall with an outstretched hand. The fracture configuration is influenced by the bone density of the proximal humerus and arm position while striking the floor. Moreover, amongst the elderly population, existing osteoporotic condition or contact against adjacent glenoid and acromion or the pull of intrinsic (rotator cuff) or extrinsic muscles (pectoralis major) during a fall can contribute to PHF.

Still, factors such as osteoporotic bone quality, the fragile soft tissue surrounding the bone, and age-related co-morbidities hinder the management of this kind of fracture [4,5].

Severely displaced three- and four-part PHF in the elderly population results from dislocations of the humeral head, head-splitting fractures, and fractures with more than 50% involvement of the humeral head [6-10].

Twenty-nine percent of the patients required hospitalization; 75% of these were over 60 years old. Only 21% of these were operated on, the majority of admissions being for social reasons.

Conservative management with a universal shoulder immobilizer or closed reduction with internal fixation using k-wire for three- or four-part PHF has poor outcomes [9 11]. Thus, they are typically managed with open reduction and internal fixation (ORIF) using any of these methods 1) proximal humeral plates, 2) hemiarthroplasty, 3) percutaneous or minimally invasive techniques such as pinning, screw osteosynthesis, and 4) the use of intramedullary nails. Satisfactory anatomical reduction and regaining functional range of motion (ROM) are critical treatment objectives for managing PHF.

The proximal humerus internal locking system (PHILOS®, Synthes, Switzerland) is the choice of implant for treating displaced or complex PHF due to its anatomically analogous design. It enables angled stabilization and improves pull-out strength in osteoporotic bone by using a locked construct of convergent and divergent screws. However, few prospective studies are available that evaluate the results of this technique or report on the treatment-related complications [9-17].

Primary hemiarthroplasty along with adequate post-operative physiotherapy has profound pain relief and improved functional outcomes. It offers a less cumbersome procedure to enable a stable fixation to achieve reduction in four-part comminuted fractures. Still, functional outcomes have been subjective [12-15].

Achieving pain-free shoulder function after PHF depends on age, medical condition, bone quality, proper evaluation of the current fixation techniques, and patient expectations. There are also surgical complications such as necrosis of the humeral head, intra-operative humerus shaft fracture, malpositioning of greater/lesser tuberosity, loosening or failure of the implant, failure of osteosynthesis, and malunion of the fracture [15]. These factors make identifying optimal treatment for the management of PHF a challenging task [5-8].

This study aims to assess the surgical outcome of two interventions viz osteosynthesis using PHILOS® plate and primary hemiarthroplasty using Neer’s prosthesis. The surgical outcome will be evaluated through functional and radiological outcomes to potentially identify the superiority of one procedure over the other for managing three- and four-part PHFs.

## Materials and methods

A prospective observational study was conducted to identify a suitable intervention for the management of PHF amongst the elderly population by evaluating post-operative functional outcomes between patients who received PHILOS® plate osteosynthesis and primary hemiarthroplasty using NEER’s prostheses.

This study was conducted in the Department of Orthopaedics at Dr. D. Y. Patil Medical College, Hospital & Research Centre, Pimpri, Pune between May 2019 to May 2021. Ethical clearance was obtained from the Meeting of Research and Recognition Committee under the faculty of medicine (IESC/PGS/2019/103). Informed consent was obtained from all participants before surgical procedures. Patients considered for the study underwent surgical procedures as per the standard guidelines. Pre- and post-operative assessments were performed to evaluate their functional outcome. Finally, statistical analysis was performed to assess the surgical outcome of PHILOS plate osteosynthesis versus primary hemiarthroplasty with Neer’s prosthesis for management of three- and four-part PHF in the elderly population.

Inclusion criteria: 1) Patients should be above fifty-five years of age; 2) patients should present within displaced proximal humerus three- or four-part fracture.

Exclusion criteria: 1) Patients below fifty-five years of age; 2) patients presenting with one- or two-part humerus fracture; 3) patients with neurological or psychiatric disorders; 4) associated rotator cuff injury; 5) non-cooperative patients for post-operative rehabilitation; 6) unwilling patients.

Pre-surgical evaluation: Patients were thoroughly assessed (both general and systemic) to characterize their injury and determine any complications and co-morbidities. The fracture was classified using Neer’s classification [8].

**Figure 1:**
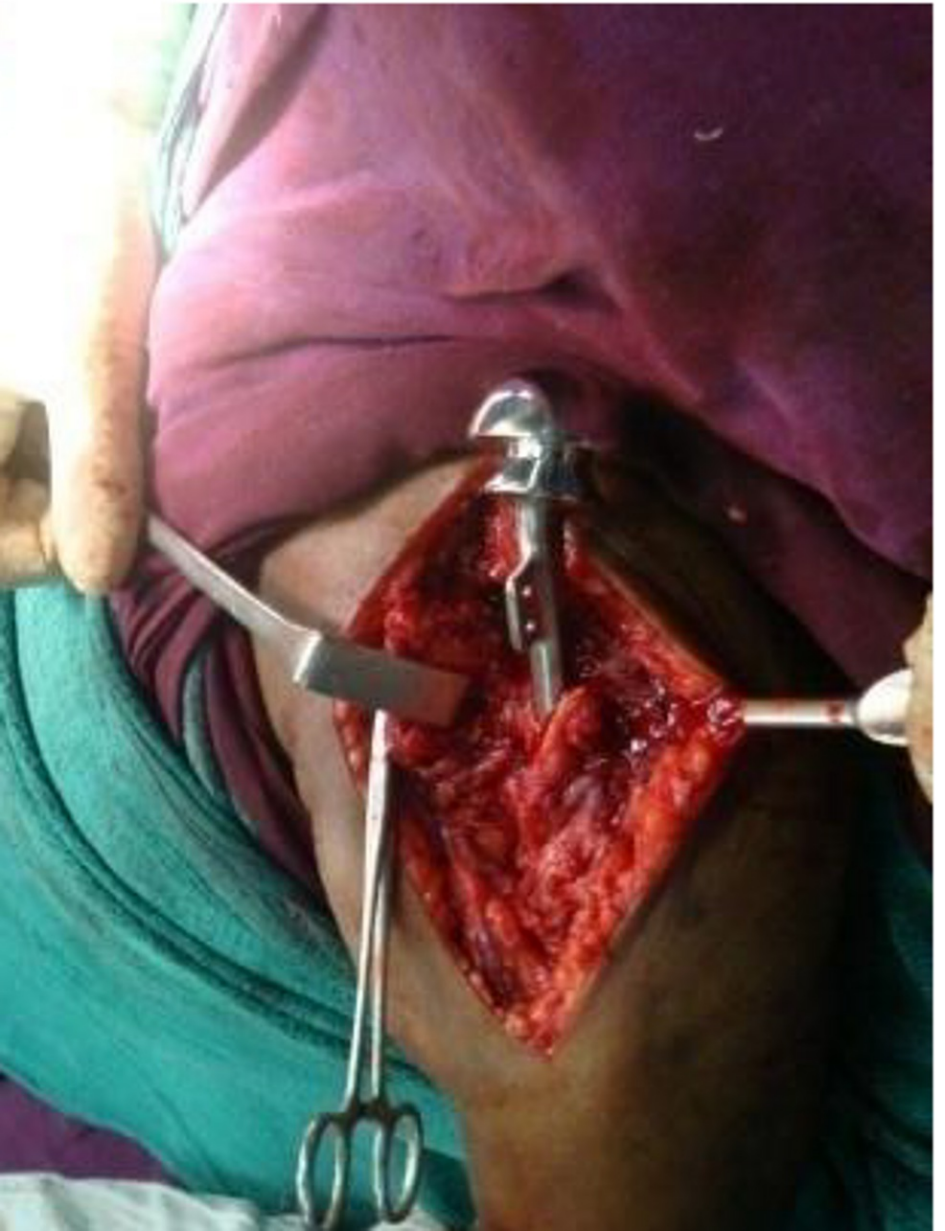
Insertion of Neer’s prosthesis; insert showing tuberosity reconstruction after Neer’s prosthesis

Surgical procedures: Patients were randomly divided into two groups of ten who received PHILOS plating and Neer’s hemiarthroplasty. Both groups underwent identical pre-operative assessment, surgical care, and evaluation protocol. Patients were put in the supine position during surgery, and a sandbag was used in the interscapular region to elevate it by 30-45 degrees. Three days of intravenous antibiotics, followed by three days of oral antibiotics, were given post-operative. Drain removal was done on the second pod and suture removal on the twelfth pod. The opted surgical method was the deltopectoral approach; wherein, an incision was made over the coracoid process and advanced along the deltopectoral groove with subsequent identification and lateral reflection of the cephalic vein. Fracture hematoma was cleared after identifying the subdeltoid space, and respective surgical interventions were performed (PHILOS® plating or Neer’s hemiarthroplasty), as seen in Figure 1. After surgery, the wound was given a thorough saline wash and was closed layer-wise with Romo vac in situ. The humerus canal was serially reamed to deliver the prosthesis (Neer’s hemiarthroplasty) by fixing the tuberosities. Here, the retroversion was set between 20 degree - 30 degree, and the prosthesis was adjusted to the appropriate anatomical length and later cemented (Figure 1).

**Figure 2:**
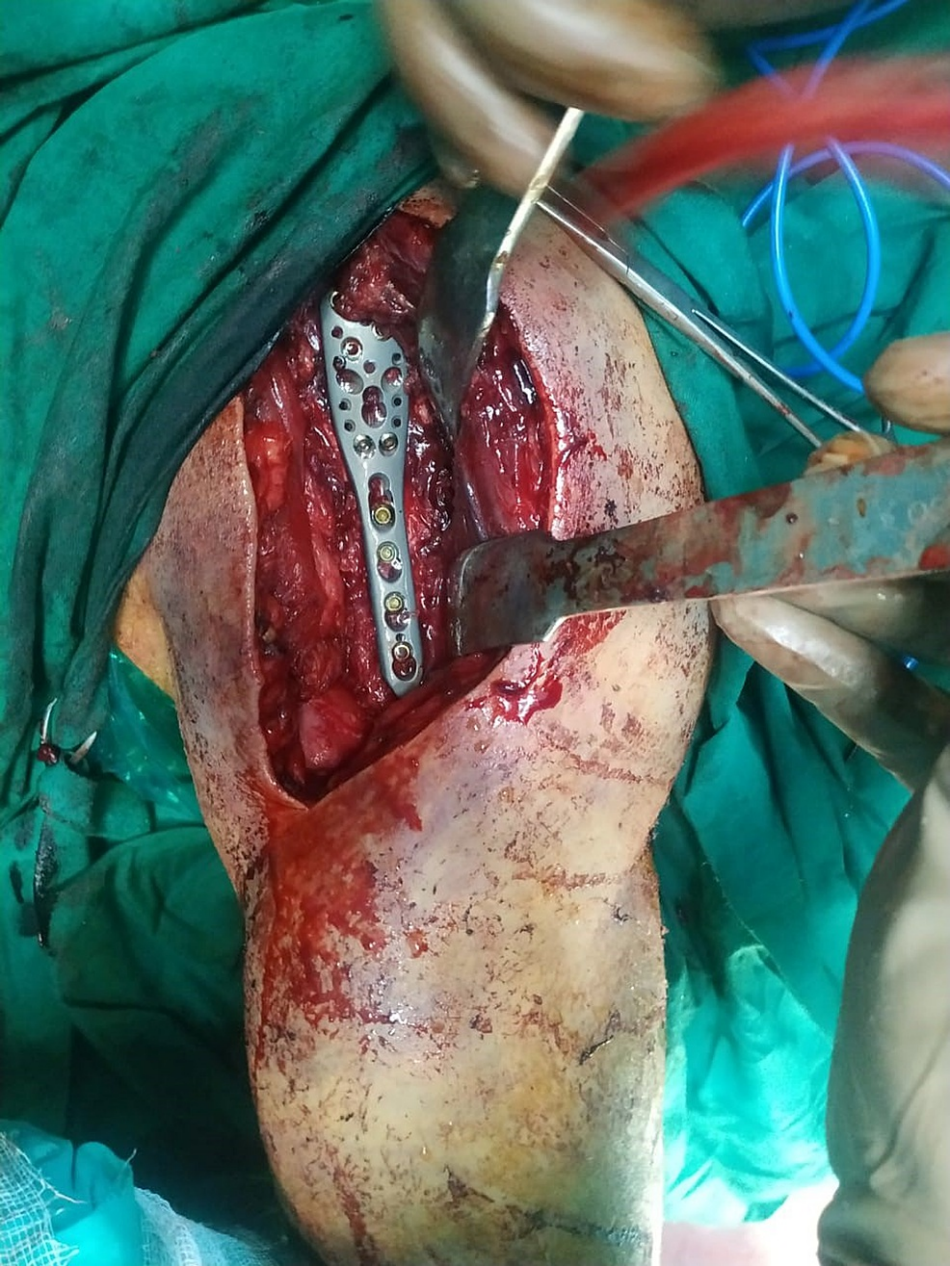
Intra-operative proximal humerus internal locking system (PHILOS®) plate fixation

**Figure 3:**
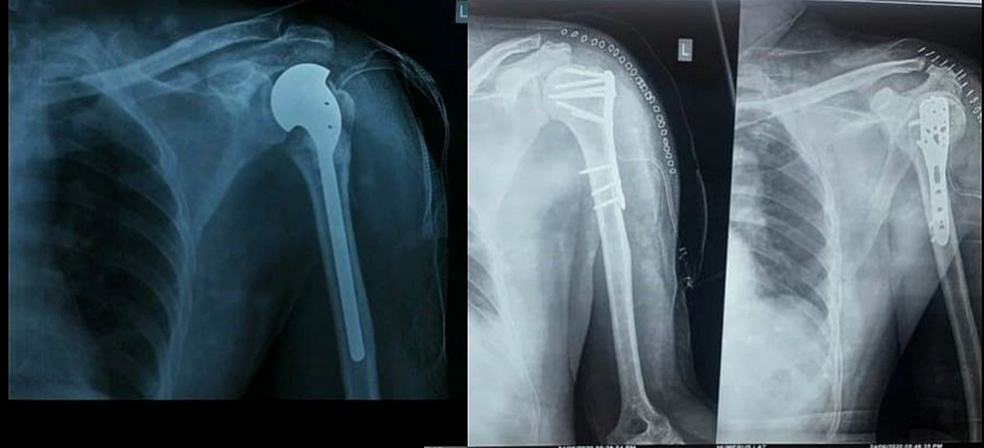
Immediate post-operative X-ray for Neer's hemiarthroplasty and proximal humerus internal locking system (PHILOS®) plating

Similarly, the surgical technique for PHILOS® plate osteosynthesis involved tuberosities and humeral head reduction along with rotor cuff tendons tagged with eithbond sutures to tie the final plating; wherein, k-wires were used for temporary fixation. PHILOS® plate was put lateral to bicipital groove and fixed with locking and cancellous screws (Figure 2). After the surgery, immediate post-operative X-ray was done which is shown in Figure 3.

Post-operative rehabilitation: Both groups received the same post-operative rehabilitation. The affected limb was immobilized using a shoulder immobilizer for the first two weeks. After the immobilization period, patients underwent Phase I exercises for two weeks to improve grip strength and elbow ROM. Phase II exercise lasted for six weeks to improve shoulder function. Lastly, isometric muscle-strengthening workouts were performed until twenty-four weeks to further return to work status.

Post-operative evaluation: Functional assessments were performed at three, six, twelve, and twenty-four weeks to evaluate the overall surgical outcome and final functional outcomes were measured on 24th post operative week. Constant Murley score and ROM for flexion, extension, abduction, internal and external rotation were used to quantify the functional outcome.

Statistical analysis: Functional effects of PHILOS® plate osteosynthesis and Neer’s hemiarthroplasty were inferred from statistical analysis performed using SPSS 17.0 software (Chicago, IL, USA). Appropriate parametric (Student’s t-test) and non-parametric tests (Fisher’s exact test) were conducted to identify significant differences in functional outcomes between the two interventions.

## Results

Twenty patients were randomly divided into two groups for PHILOS® plating and Neer’s hemiarthroplasty.

Table 1 summarizes the patient characteristics. The average age for the PHILOS® plating group was 67.2 years, whereas, for Neer’s hemiarthroplasty, it was 72.8 years. Five males and five females underwent Neer’s hemiarthroplasty, and three females and seven males underwent PHILOS® plating. Based on their occupation, there was a carpenter (1), driver (1), farmer (3), homemaker (7), painter (1), plumber (1), retired (5), and salesman (1). For both these interventions, surgery was done on the left side for six patients and on the right side for four patients.

**Table 1:** Patient characteristics for proximal humerus internal locking system (PHILOS) plating and Neer's prosthesis interventions

Intervention	Mode of injury		Gender		Total	Age
	Fall	Road traffic accident	Male	Female		
NEER	5	5	5	5	10	72.8
PHILOS^®^	5	5	7	3	10	67.2

During the intra-operative assessment, the average blood loss in PHILOS® plating was 308 ml, while Neer’s hemiarthroplasty was 341 ml. Also, the average surgery duration was 68.5 min and 92.5 min, respectively. Based on the amount of blood loss and surgery duration, PHILOS® osteosynthesis was found to be better than Neer’s hemiarthroplasty.

Functional and radiological outcomes were evaluated post-operatively on out patient follow-up basis and final functional outcomes were measured and compared on 24th post operative week. Accordingly, the shoulder ROM was assessed for flexion, extension, abduction, and internal and external rotation, compared between the two groups as in Table 2.

**Table 2:** Comparison of range of motion between proximal humerus internal locking system (PHILOS) plating and Neer's prosthesis interventions p-value <=0.05 indicates a statistically significant difference between the groups compared.

Movement	Technique		Mean	p-value
Flexion		NEER	89.5	0.0218
		PHILOS^®^	109.5	
Extension		NEER	44	0.0246
		PHILOS^®^	49.5	
Abduction		NEER	85	0.0304
		PHILOS^®^	97.5	
Internal roation		NEER	52	0.0311
		PHILOS^®^	58	
External rotation		NEER	54	0.0444
		PHILOS^®^	64	

Similarly, the Constant-Murley score quantified the shoulder function on 24th post operative week (Table 3).

**Table 3:** Comparison of Constant-Murley score between proximal humerus internal locking system (PHILOS) plating and Neer's prosthesis interventions p-value <=0.05 indicates a statistically significant difference between the groups compared.

Constant Murley score	Technique	Mean	p-value
	NEER	62.6	0.0267
	PHILOS^®^	71.3	

A statistically significant p-value (p < 0.05) was observed for these comparisons.

The average degree of flexion for individuals treated with Neer’s hemiarthroplasty was 89.5^o^, whereas the same for the PHILOS® group was 109.5^0^, which was 20^0^ more in the PHILOS osteosynthesis group. With a 20% increase, patients who received PHILOS® osteosynthesis had better flexion.

The average degree of extension for individuals treated with Neer’s hemiarthroplasty was 44^o^, whereas the same for the PHILOS® group was 49.5^0^, which was 5.5^0^ more in the PHILOS osteosynthesis group. With a 12.5% increase, patients who received PHILOS® osteosynthesis had a better extension.

The average degree of abduction for individuals treated with Neer’s hemiarthroplasty was 85^o^, whereas the same for the PHILOS® group was 97.5^0^, which was 12.5^0^ more in the PHILOS osteosynthesis group. With a 14.7% increase, patients who received PHILOS® osteosynthesis had a better abduction.

The average degree of internal rotation for individuals treated with Neer’s hemiarthroplasty was 52^o^, whereas the same for the PHILOS® group was 58^0^, which was 6^0^ more in the PHILOS osteosynthesis group. With an 11.5% increase, patients who received PHILOS® osteosynthesis had a better internal rotation.

The average degree of external rotation for individuals treated with Neer’s hemiarthroplasty was 54^o^, whereas the same for the PHILOS® group was 64^0^, which was 10^0^ more in the PHILOS osteosynthesis group. With an 18.5% increase, patients who received PHILOS® osteosynthesis had a better external rotation.

The average constant Murley score for individuals treated with Neer’s hemiarthroplasty was 62.6, whereas the same for the PHILOS® group was 71.3, which was 8.7 units more in the PHILOS osteosynthesis group. With a 15% increase, patients who received PHILOS® osteosynthesis had a constant Murley score.

**Figure 4:**
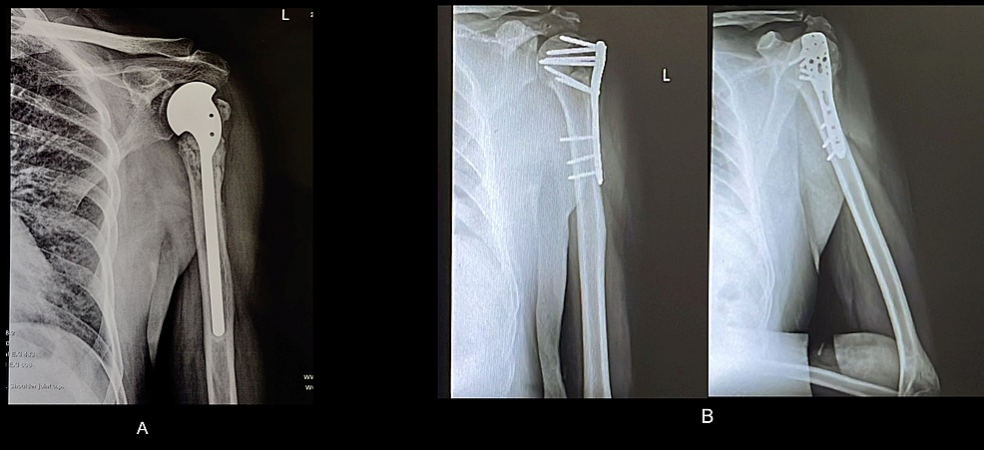
Two years post-operative follow-up X-ray of Neer's hemiarthroplasty and proximal humerus internal locking system (PHILOS) plating

Post-operative X-ray were done and were repeated on OPD follow-up (Figure 4).

**Figure 5:**
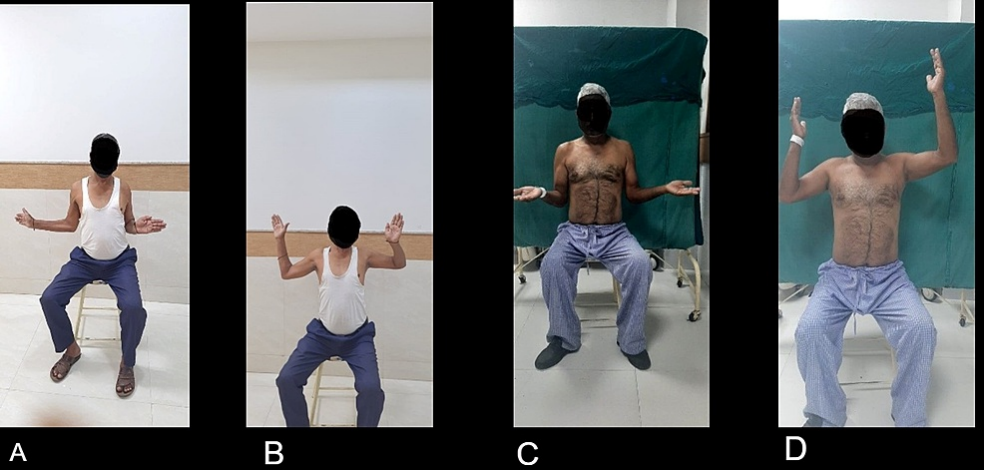
Figures A & B represents functional outcome of Neer's hemiarthroplasty and Figure C & D represents functional outcome after proximal humerus internal locking system (PHILOS) plate osteosynthesis

Radiological assessment was done on sequential follow-up. Most of the above cases were united radiologically, and the prosthesis was well reduced in anatomical position. One case of Neer’s showed aseptic loosening, and one had superior migration on follow-up X-ray. One case of PHILOS osteosynthesis showed non-union on X-ray. The critical advantage of PHILOS osteosynthesis over Neer’s hemiarthroplasty was better post-operative ROM with routine physiotherapy protocol, whereas Neer’s hemiarthroplasty required more rigorous physiotherapy to avoid post operative stiffness and capacity to perform daily activities. Post-operative ROM following the two interventions is shown in Figure 5.

## Discussion

Three- and four-part PHF in elderly population can significantly impact their quality of life. Moreover, this demographic is prone to osteoporosis and tends to have PHF from a simple fall. Aiming to find a suitable intervention for managing three- and four-part PHF in the elderly population, this study compared the functional outcomes of two commonly used techniques, PHILOS® plate osteosynthesis, and Neer’s hemiarthroplasty.

Overall clinical outcome using the Constant Murley-score was higher for the PHILOS® group (71.3). A similar improvement in functional outcome was reported across similar studies with PHILOS® plating. Despite the brief duration for follow-ups in our study, investigations have shown that early function improvement has long-term outcomes. The prognosis appears to be linked to the implant’s proper placement and fixation. Moreover, suturing the tendons within the aperture of the locking plate lowed the likelihood of implant failure. In conjunction with complete anatomical reduction, precise plate placement resulted in a marked improvement in functional results. This was seen with decreased Constant-Murley scores when anatomical reconstruction failed or when a non-anatomical reconstruction was accepted intra-operatively, or when the plate was not correctly positioned on the shaft at the appropriate height to avoid subacromial impingement. Also, there is no statistically significant difference between screw perforation in the joint and acute infection.

A consequence of ORIF is the perforation of the humeral head by a lengthy locking screw. However, the proportion of screw pull-out was significantly reduced with PHILOS® plating due to the locking head and set angle orientation. The multidirectional nature of the screws in the locking plate covered the entire sphericity of the head rather than the center alone. This decreased humeral head fixation failure and its collapse. The prevalence of losing the vascularity of the humeral head was also less. A thorough fluoroscopic examination was performed after the screw was installed to ensure that the humeral head was not perforated due to an incorrect screw length.

Another common consequence of PHILOS® plate osteosynthesis was osteonecrosis. Depending on the osteosynthesis process, there is a fifty-percent chance of avascular necrosis of the humeral head. Nevertheless, even patients who developed osteonecrosis had a fair functional outcome at the early follow-up, comparable to patients who received Neer’s hemiarthroplasty.

Patients with the humerus head in valgus position had a better outcome than those with a varus impacted fracture. This was because the PHILOS® plate acted as mechanical support under a compressive force that resisted valgus collapse. On the contrary, the plate acted as a tension band that pulled the humeral head out when the head was in a varus position.

Due to the osteoporotic nature of aged bone, varus fractures significantly disfavored the implant mechanically. And implant failure was due to screw pull-out resistance rather than bone compressive strength. Additionally, the success of PHILOS® plate osteosynthesis could be that the rotator cuff tendon was not directly manipulated during reduction and internal fixation. To eliminate confounding bias for constant score, patients with a rotator cuff injury, either previously documented or diagnosed intra-operatively, were excluded from the study.

In summary, the ROM pertaining to flexion, extension, abduction, internal rotation, and external rotation for individuals with PHILOS® plating was 20%, 12.5%, 14.7%, 11.5%, and 18.5% higher than those who received Neer’s hemiarthroplasty. Moreover, the Murley score was also 8.7 units higher for individuals with PHILOS® plating. The intra-operative assessment parameters (blood loss and surgery duration) also favored PHILOS® plate osteosynthesis.

## Conclusions

This study aimed to identify a suitable intervention for the management of PHF. Accordingly, a prospective observational study was conducted to evaluate the surgical outcome when considering PHILOS® plate osteosynthesis and primary hemiarthroplasty using Neer’s prostheses. Although hemiarthroplasty yielded a consistent functional outcome, PHILOS® plate osteosynthesis tends to restore a greater ROM and had a better overall outcome than hemiarthroplasty with Neer’s prosthesis for the management of three- and four-part PHF for people above fifty-five years of age.
